# Using patient flow analysis with real-time patient tracking to optimize radiation oncology consultation visits

**DOI:** 10.1186/s12913-022-08809-2

**Published:** 2022-12-13

**Authors:** Shane Mesko, Julius Weng, Prajnan Das, Albert C. Koong, Joseph M. Herman, Dorothy Elrod-Joplin, Ashley Kerr, Thomas Aloia, John Frenzel, Katy E. French, Wendi Martinez, Iris Recinos, Abdulaziz Alshaikh, Utpala Daftary, Amy C. Moreno, Quynh-Nhu Nguyen

**Affiliations:** 1 Scripps MD Anderson Cancer Center, Division of Radiation Oncology, San Diego, California USA; 2 Department of Radiation Oncology, MD Anderson Cancer Center, Houston, TX USA; 3 Institute for Cancer Care Innovation, MD Anderson Cancer Center, Houston, TX USA; 4 Chair, Patient Informatics, MD Anderson Cancer Center, Houston, TX USA

**Keywords:** Patient flow analysis, Clinical efficiency, Clinical workflow, Cycle time, Rooming time, Waiting time, Radiation oncology

## Abstract

**Purpose:**

Clinical efficiency is a key component of the value-based care model and a driver of patient satisfaction. The purpose of this study was to identify and address inefficiencies at a high-volume radiation oncology clinic.

**Methods and materials:**

Patient flow analysis (PFA) was used to create process maps and optimize the workflow of consultation visits in a gastrointestinal radiation oncology clinic at a large academic cancer center. Metrics such as cycle times, waiting times, and rooming times were assessed by using a real-time patient status function in the electronic medical record for 556 consults and compared between before vs after implementation of the PFA recommendations.

**Results:**

The initial PFA revealed four inefficiencies: (1) protracted rooming time, (2) inefficient communications, (3) duplicated tasks, and (4) ambiguous clinical roles. We analyzed 485 consult-visits before the PFA and 71 after the PFA. The PFA recommendations led to reductions in overall median cycle time by 21% (91 min vs 72 min, *p* < 0.001), in cumulative waiting times by 64% (45 min vs 16 min; *p* < 0.001), which included waiting room time (14 min vs 5 min; *p* < 0.001) and wait for physician (20 min vs. 6 min; *p* < 0.001). Slightly less than one-quarter (22%) of consult visits before the PFA lasted > 2 h vs. 0% after implementation of the recommendations (*p* < 0.001). Similarly, the proportion of visits requiring < 1 h was 16% before PFA vs 34% afterward (*p* < 0.001).

**Conclusions:**

PFA can be used to identify clinical inefficiencies and optimize workflows in radiation oncology consultation clinics, and implementing their findings can significantly improve cycle times and waiting times. Potential downstream effects of these interventions include improved patient experience, decreased staff burnout, financial savings, and opportunities for expanding clinical capacity.

## Introduction

Healthcare quality and value are critical components of the patient-centric care model and are increasingly tied to reimbursement of medical care. The US Institute of Medicine has developed a framework of six domains for measuring health care quality, two of which are “timely” and “efficient” care. These two components may be even more important for patients diagnosed with cancer: for those with early-stage malignancies, interactions with the healthcare system consume up to one-third of the first 60 days of treatment and 57 days over the next 18 months [1, 2]. For cancers with very poor prognoses, this can mean that as many as 10% of patients’ total days of survival involve a health care encounter [3]. Further, the median time per visit for these encounters is as high as 4.6 h, a considerable portion of which is spent waiting for care [1, 3].

Not surprisingly, long wait times are widely cited as a key source of patient frustration, especially among oncology patients [4–6]. This frustration is shared by physicians and support staff, who collectively cite subjective time pressure and administrative burden as frequent sources of burnout [7]. Studies of radiation oncology patient satisfaction in particular have supported this notion, with both wait times and patient education cited as key influencers of patient satisfaction [8]. Considering the large number of visits faced by patients with a cancer diagnosis, small reductions in wait times and overall cycle times for each visit can have a profound cumulative impact.

As the number of new diagnoses and the complexity of cancer care continue to grow, processes aimed at improving clinical efficiency provide a rare opportunity for alignment of the goals of patients, providers, and hospital administration. One approach to the challenge of providing more timely and efficient care is patient flow analysis (PFA), in which patients are tracked through every step of a visit to identify inefficiencies in the workflow. In this study, we proposed a PFA for radiation oncology consultation visits at a large, multidisciplinary cancer center. We then used the findings from this initial analysis to optimize the clinic workflow and measured the resulting effects on consultation cycle times and waiting times.

## Methods

A quality improvement protocol was designed for patients with consult appointments in the Department of Gastrointestinal Radiation Oncology at The University of Texas MD Anderson Cancer Center, a large US tertiary cancer care center. The study was conceived in February 2019 and was approved by the institutional quality improvement approval board. The Department of GI radiation oncology includes 7–9 faculty-level physicians or physicists specializing in radiation oncology and a collective volume of more than 750 consults in a typical year. Consultations are held in a dedicated clinical space with 3–4 exam rooms. Each consult clinic was staffed by one attending physician at a time. The clinical support staff typically included an advanced practice provider, a radiation oncology resident or fellow, 1 or 2 registered nurses, and, when available, a medical assistant.

### Patient flow analysis

The first step was to use PFA to assess the existing workflow and establish a baseline for comparison. PFA is a broadly replicable practice by which the entire care process is outlined, from the patient perspective, to identify inefficiencies or bottlenecks and develop potential solutions [9–11]. The PFA involves quantifying the amount of time spent at each step, identifying which staff members are involved, and describing the specific tasks performed during each component of the visit. This initial phase took place from February 2019 through May 2019 and included verification of time stamp data from electronic medical record (EMR) using in-person observations. This preliminary data were subsequently reviewed, and the resulting findings were summarized and discussed with key stakeholders (nursing, physicians, department leadership, and administrative staff) to identify opportunities for improvement. Recommendations for the planned interventions to the clinical workflow were agreed upon and baseline data was collected from May 2019 to December 2019.

The new workflow (described in the Results section) was piloted for one physician’s clinical consults starting in December 2019, and was extended to the entire radiation oncology GI service in February 2020. The implementation phase and subsequent data collection were unfortunately interrupted by the COVID-19 pandemic, which necessitated new protocols for room cleaning, use of personal protective equipment, reductions in waiting room capacity, and staffing restrictions. These factors, and the temporary decline in patient volumes and operations, delayed the collection of post-implementation findings, which began in July 2020 and ended in September 2020.

### Outcome measures

**Fig. 1:**
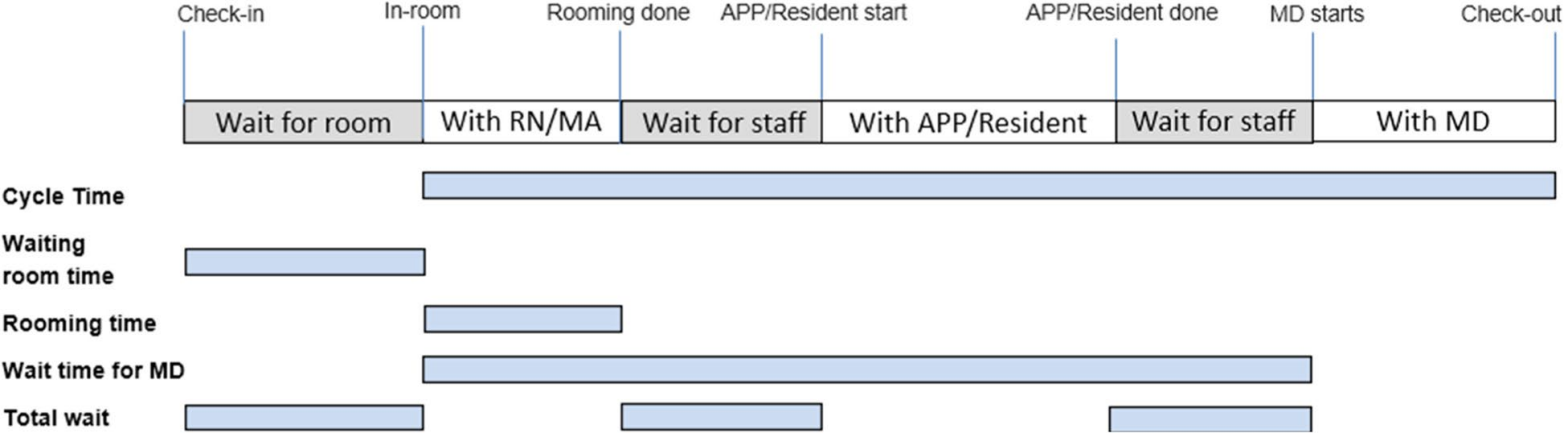
Schematic of the various steps constituting a consultation visit in radiation oncology at the authors’ institution. At left are the metrics of clinical efficiency to be examined in the patient flow analysis. Metrics were compiled from documentation of patient status from the electronic medical record. Abbreviations: APP, advanced practice provider; MD, physician; RN/MA, registered nurse/medical assistant

The EMR (Epic Systems; Verona, WI, USA) was used to augment in-person data collection regarding the timing and staff involved in each step in the process. At the authors’ institution, this software includes a real-time “Patient Status” function, where the patient’s location and interactions with staff can be recorded throughout the consultation process. Staff were educated on the status board functionality before the project was launched, and the EMR data were cross-referenced with in-person data for validation. Notably, not every data point (e.g., every status) was captured for every patient. Time points lacking data (whether from the EMR) were censored from the corresponding metric of clinical efficiency (Fig. 1). Data were periodically reviewed by hand for erroneous or missing entries (e.g., staff forgetting to select “check out” until the following day).

Metrics of clinical efficiency (that is, the entire cycle time [from in-room to check-out]; waiting room time [from arrival to in-room]; rooming time [time spent settling the patient in the exam room, obtaining vital signs, and basic screening]; wait for the physician [from in-room to physician arrival]; and total wait time [intervals that patients spent waiting throughout the entire process], Fig. 1) were measured and compared from before the PFA to afterward. Time spent with advanced practice providers, residents, and attending physicians was also measured and included in the metric ‘cycle time’.

These metrics were then stratified by physician for in-depth comparisons. Statistical analyses were done with SPSS (Version 25.0, IBM, Armonk, NY). Mann–Whitney U tests were used to compare non-parametric data, which were reported as medians and interquartile ranges (IQR; 25^th^-75^th^ percentile). Chi-squared analysis was used for proportional comparisons.

## Results

### Baseline findings

**Fig. 2:**
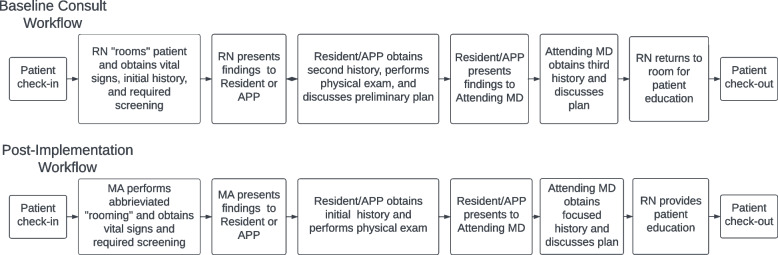
Process maps from the initial patient flow analysis and the patient flow analysis after implementation of the recommended modifications. Abbreviations: RN, registered nurse; APP, advanced practice provider; MA, medical assistant; MD, physician

Process maps were created during the first phase of initial data collection depicting the existing workflow (Fig. 2). These maps and observations revealed several key findings. First, the rooming process was taking longer than anticipated, often because of required screenings or assessments and the need to obtain brief patient histories. This was compounded by the intermittent availability of a second nurse, often leading to ambiguity over who was responsible for which tasks (e.g., “who is rooming the next patient?”). Next was duplication of effort that added to the total consultation times. For example, the nurse would take a brief patient history and present this information to the advanced practice provider or resident physician, who would then obtain a more detailed history and perform a physical exam (the classic "history & physical” or H&P). The findings were then presented to the attending physician, who would repeat components of the H&P before proceeding to providing recommendations for treatment. Also, a nurse was required to enter the room twice, once during the initial rooming process and again at the end of the visit for education for patients who are recommend radiation therapy. Communication inefficiencies also resulted in excessive in-room wait times between each staff member, especially when several patients were roomed simultaneously. Finally, review of the individual tasks being performed showed that staff were not “working to the top of their licensing or training,” i.e., Nurses were spending time on tasks (initial patient rooming) that could be executed by medical assistants.

### Recommendations

First, a revised workflow was proposed to better incorporate the role of medical assistants for basic rooming (vital signs and basic screenings), which would allow the RN to focus on education and nursing-level assessments. The proposal also shifted the RN to the end of the visit to minimize the number of times the RN was to enter and leave the room (and the resulting delays) and to allow patient education after the final plan had been established by the physician. We emphasized reducing duplicated work and task overlap by defining the roles of each staff member with respect to their training/licensing. Finally, better use of the real-time status board in the EMR was implemented to improve communication among staff members. The primary goal of these interventions was to reduce the median cycle time (that is, from the patient entering the exam room to check-out) by at least 15 min.

### Final outcomes

**Fig. 3:**
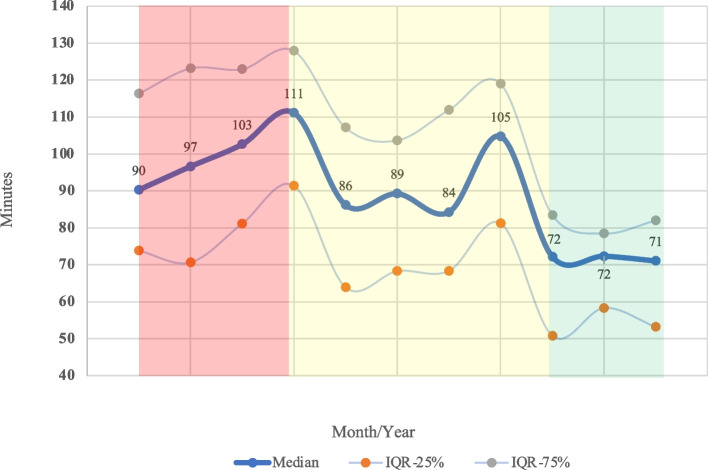
Overall cycle times (that is the intervals between the patient entering the exam room until check-out), in minutes, over the course of the study period. The thick line indicates median times and the thinner lines the interquartile range (25^th^ to 75^th^ percentile). The shaded area at left represents the initial observation phase (May–August 2019); the middle shaded area indicates when the baseline data were discussed and recommendations were formulated (August 2019–July 2020); and the shaded area at right is after implementation of the recommendations from the patient flow analysis

We analyzed findings from 485 patient visits during the 7-month period before implementation of the PFA recommendations and 71 patient visits afterward (from July 2020 through September 2020). Overall, the median cycle time was reduced by 21% (absolute reduction of 19 min), from 91 to 72 min (IQR 71–114 min before vs 52–82 min after, *p* < 0.001), which met the primary goal of a 15-min reduction. Roughly one-quarter of consultation visits (22%) before implementation lasted > 2 h as compared with 0% after implementation (*p* < 0.001). Similarly, the proportion of visits completed in < 1 h was 16% before vs 34% after implementation. Cumulative median waiting times (i.e., ‘Total Wait’ in Fig. 1) were reduced by 64% (median 45 min vs 16 min, *p* < 0.001). Patients also spent 64% less time in the waiting room (median 14 min vs 5 min, *p* < 0.001) despite no significant changes in the proportion of patients arriving > 15 min early (43.9% vs 49.3%, *p* = 0.393), within 15 min of the appointment time (43.9% vs 33.8%, *p* = 0.109), or > 15 min late (12.2% vs 16.9%, *p* = 0.269). Seven physicians saw patients in both the pre- and post-implementation phases, and all experienced improved mean cycle times (17%-46% reduction, or an absolute reduction of 13–44 min per visit). All other components of the consult visit were either significantly reduced or maintained (Table 1). A graph showing the median/IQR cycle times at each phase of the project is shown in Fig. 3. In terms of patient volume, the average number of consults per day was 5.0 before vs 3.1 after implementation. However, video consults were implemented in the post-COVID-19 era (*n* = 26) and were not included in this project because of the differences in workflow. However, given that video consults took place during the same clinic time as standard consults, factoring in the video consults brought the daily average number of consults to 4.2 after implementation of the PFA recommendations.

**Table 1:** Metrics of clinical workflow before and after a patient flow analysis

**Metric**	**Time before PFA, min,** **median (IQR)**	**Time after PFA, min,** **median (IQR)**	**Delta**	***P*** Value*
Waiting room	14 (8–26)	5 (3–14)	–64%	< 0.001
Arrived > 15 min early	20 (11–41)	10 (4–20)	–50%	< 0.001
Arrived within 15 min	12 (7–19)	4 (3–12)	–67%	< 0.001
Arrived > 15 min late	8 (5–15)	2 (1–8)	–75%	< 0.001
Rooming (RN/MA)	13 (9–18)	12 (9–14)	–8%	0.066
Waiting for APP/Resident	11 (5–20)	5 (3–8)	–55%	< 0.001
With APP/Resident	22 (12–32)	19 (12–26)	-14%	0.490
Waiting for MD	20 (11–33)	6 (3–15)	–70%	< 0.001
With MD	33 (25–48)	23 (15–31)	–30%	< 0.001
Time in-room to arrival of MD	54 (39–72)	47 (33–60)	–13%	0.003
Total cycle time	91 (71–114)	72 (52–82)	–21%	< 0.001

“Rooming” refers to settling a patient in an exam room and obtaining vital signs and initial screening

*Abbreviations: PFA* Patient flow analysis, *IQR* Interquartile range (25^th^ – 75^th^ percentiles), *RN* Registered nurse, *MA* Medical assistant, *APP* Advanced practice provider, *MD* Physician

^*^ Calculated with Mann–Whitney U test

## Discussion

Timely, efficient care is important in the treatment of patients diagnosed with cancer, who often spend a significant proportion of their time interacting with the healthcare system. In this study, we demonstrated that patient flow analysis was an excellent tool for revealing inefficiencies in the workflow and subsequently optimizing the workflow of consultations in a high-volume radiation oncology center. Specifically, we found subpar communications, duplicated tasks, and ambiguous staffing roles to be the greatest impediments to an efficient process. Our recommendations and workflow changes led to significant improvements in overall consult visit efficiency and waiting time for patients.

For treatment centers looking to improve their clinical efficiency, PFA is broadly applicable in various settings and can often be done within the structure of existing clinics, without substantial cost and with minimal disruption to existing operations. Combining PFA with real-time patient status data through the EMR allowed us to rapidly collect a large number of granular data points, and presumably automating this process could provide such data on an ongoing basis. A similar real-time patient tracking study of 84 radiation oncology patients at Johns Hopkins found an average consult cycle time of 89.4 min, with 57% of the time (51.2 min) spent waiting in the exam room [12]. These findings are very similar to our baseline cycle time (median 92 min) and our wait time of 45 min. The authors of the Hopkins study did not implement a process change but rather used two methods to document workflow and identify one source of the inefficiencies that were noted. Interestingly, this model resulted in a potential cycle time of 65.3 min with 27.1-min wait times, which are also quite similar to our final outcomes. Although we focused only on consult visits, the Hopkins study also modeled potential improvements for follow-ups, “weekly see” visits, and nurse visits. These findings, and their similarity to ours in the current study, suggests that broad-scale process improvements could be implemented by using PFA.

The overall improvements in cycle time in our study were likely driven by several factors. For example, even though our EMR real-time status system is invaluable for data collection, perhaps more important is its function as a communication tool to inform staff of where each patient is in the workflow. Better use of this function likely contributed to a large proportion of the reduction in cycle time coming from decreased wait times between staff visits, which probably reflects better communications regarding patient status and a more clearly defined workflow. These are crucial points, because wait times drive patient dissatisfaction, and “face time” with a physician drives satisfaction [5, 13, 14]. This point is also particularly germane in an era in which burnout is becoming endemic among health care providers, perhaps leading to staffing shortages. In our study, better awareness of clinical roles also reduced the redundancies in the process, leading to small but significant improvements. Beyond the absolute reduction in time, the interquartile ranges were also substantially narrowed. The cut-off for the longest 25% of consults was 114 min at baseline vs 82 min after implementation; this finding could lead to the development of more consistently predictable scheduling templates. Finally, the Hawthorne effect, which postulates that subjects may change their behavior because they are aware that they are being observed, should be considered in interpreting the findings from this study. For example, the decrease in cycle times during the discussion phase (middle panel of Fig. 3), before any process changes had been implemented, suggests that the staff being aware that the process was being observed may have contributed to part of that reduction in cycle time.

Although this study met its primary goal of reducing cycle times, additional opportunities exist to improve the patient experience and reduce visit/wait times still further. For example, simple interventions like sending patients an educational video before the radiotherapy consultation has been shown to improve the education process and the efficiency of in-clinic discussions [15]. Virtual care platforms could also be expanded to improve the pre- and post-consult experience by shifting the administrative, low-value components of the visit to a more comfortable patient setting that does not require travel to the clinic and its associated costs. As alluded to previously, these findings also have implications for provider burnout, shown recently to be experienced by up to 56% of radiation oncology clinical staff, with reported drivers including workload control, job stress, inadequate time to document, and EMR time spent at home [16]. Reducing the time spent in clinic through reducing cycle time and minimizing disruptions to the scheduling process may improve these and other job-related stress factors.

Using EMR data allowed us to collect a larger and perhaps more robust number of patient data points than would have been possible through solely in-person observation; nevertheless, several additional limitations remain to be addressed. First, this study was conducted in a subspecialty service at a large single institution. Even though the PFA process can be generalized to many settings, our findings and proposed solutions may be unique to our practice; nevertheless, best practices may exist that can be shared to accelerate the process in future implementations. Second, the EMR status data were not comprehensive for every patient. Because this step relies on the staff updating the patient status in real time, some timepoints may have been missed at times the clinic was too busy or the staff could not access the EMR promptly. Because missing data elements may be more common during busy clinics, cycle times may be underestimated. However, this factor may be balanced by subtle overestimates at each time point, given the likelihood of a small delay between EMR status updates and the actual events. Moreover, the effects of the COVID-19 pandemic are difficult to model. The small reduction in average number of consults per day in our study (from 5 to 4.2) undoubtedly reflected the addition of protective measures, such as personal protective equipment, clinic room cleaning, and other staff precautions that may prolong cycle times. Finally, implementation of the new evaluation and management billing system by the Centers for Medicaid and Medicare Services in early 2021 would be expected to complicate future comparisons that span more than one coding period.

## Conclusions

Patient flow analysis can be useful for identifying clinical bottlenecks that may affect the ability to deliver timely and efficient care. Using the information gained from these analyses to optimize workflows can result in substantial reductions in wait times and overall consultation cycle times. Future efforts will focus on applying this concept to a broader population in our ambulatory care enterprise, measuring the resulting financial implications and effects on patient satisfaction, and periodically reassessing measurables to validate the durability of the improvement.

## Data Availability

The datasets generated and analyzed during the current study are not publicly available due to institutional restrictions but are available from the corresponding author on reasonable request and with permission from the University of Texas MD Anderson Cancer Center.
